# Manufacturing Process Affects Coagulation Kinetics of Ortho-R, an Injectable Chitosan–Platelet-Rich Plasma Biomaterial for Tissue Repair

**DOI:** 10.3390/bioengineering11090929

**Published:** 2024-09-17

**Authors:** Anik Chevrier, Marc Lavertu

**Affiliations:** 1 Department of Chemical Engineering, Polytechnique Montreal, 2500 Chem. de Polytechnique, Montreal, QC H3T 1J4, Canada; a.chevrier@polymtl.ca; 2 Institute of Biomedical Engineering, Polytechnique Montreal, 2500 Chem. de Polytechnique, Montreal, QC H3T 1J4, Canada

**Keywords:** chitosan, platelet-rich plasma, thromboelastography, manufacturing, bioengineering, tissue repair, biomaterials

## Abstract

Ortho-R (ChitogenX Inc., Kirkland, QC, Canada) is an injectable combination drug–biologic product that is used as an adjunct to augment the standard of care for the surgical repair of soft tissues. The drug product comprises lyophilized chitosan, trehalose and calcium chloride, and it is dissolved in platelet-rich plasma (PRP), a blood-derived biologic, prior to injection at the surgical site where it will coagulate. The first step of the Ortho-R manufacturing process involves dissolving the chitosan in hydrochloric acid. The purpose of this study was to investigate the effect of increasing the amount of acid used to dissolve the chitosan on final drug product performance, more specifically, on the chitosan–PRP coagulation kinetics. Chitosans were solubilized in hydrochloric acid, with concentrations adjusted to obtain between 60% and 95% protonation of the chitosan amino groups. Freeze-dried Ortho-R was solubilized with PRP, and coagulation was assessed using thromboelastography (TEG). The clotted mixtures were observed with histology. Clot reaction time (TEG R) increased and clot maximal amplitude (TEG MA) decreased with protonation levels as pH decreased. Chitosan distribution was homogeneous in chitosan–PRP clots at the lowest protonation levels, but it accumulated toward the surface of the clots at the highest protonation levels as pH decreased. These changes in coagulation kinetics, clot strength and chitosan distribution induced by high protonation of the chitosan amino groups were partially reversed by adding sodium hydroxide to the dissolved chitosan component in order to decrease pH. Careful control of manufacturing processes is critical, and it is important to consider the impact of each manufacturing step on product performance.

## 1. Introduction

Ortho-R (ChitogenX Inc., Kirkland, QC, Canada) is a combination drug–biologic product that comprises a lyophilized chitosan (CS)/trehalose/calcium chloride (CaCl_2_) formulation that is solubilized in autologous platelet-rich plasma (PRP) and injected into the body where it solidifies in situ [1,2,3]. It is intended to be used as an adjunct to augment the standard of care for surgical soft tissue repair. Pre-clinical studies have shown its beneficial effects on the healing of soft tissues such as cartilage [4], meniscus [5] and rotator cuff tendons [6,7,8,9], and it is currently undergoing clinical investigation in a multi-centre U.S.-based rotator cuff repair randomized controlled trial.

During the research and development phase, we produced and tested fine powders of chitosan of different molar masses and degrees of deacetylation (DDA) and found that chitosans with number average molar mass (*M*_n_) between 32 and 55 kDa and DDA between 80 and 86% met product specifications [1]. Products containing chitosans of lower *M*_n_ solidify very slowly, which causes phase separation of the chitosan and PRP components in the hybrid CS-PRP clots, while products containing chitosans of higher *M*_n_ are not soluble in PRP [1]. Products containing chitosans with higher DDA induce agglutination of the erythrocytes present in the PRP component, so handling the product becomes difficult or even impossible [2]. The manufacturing process of Ortho-R involves solubilizing the chitosan component in a hydrochloric acid (HCl) solution and mixing with stock trehalose (acting as a lyoprotectant) and CaCl_2_ (acting as a PRP solidification agent) solutions prior to freeze-drying in glass vials. We elected to solubilize the chitosan in the mildest acidic conditions possible in order to keep the pH as close to physiological as possible. We found that adding just enough HCl so that 60% of the amino groups present on the glucosamine units of the chitosan chains are protonated allows the chitosan to become soluble while maintaining the pH of the solution around 6.2. All of our early development and pre-clinical work was performed with chitosan product dissolved under these conditions [1,2,3,4,5,6,7,8].

Manufacturing of the product was then transferred to a contract manufacturing organization (CMO) (KABS Laboratories, Saint-Hubert, QC, Canada) and scaled up for production of an Ortho-R lot to be used for a pivotal sheep rotator cuff repair study [9] that would become the cornerstone of an IND application to the Food and Drug Administration (FDA). One issue that the CMO faced during scale-up was that the chitosan material generated in the form of coarse flakes could not easily be ground/milled to form a fine powder, as we had been doing on small batches during the research and development phase. The solubilization of the chitosan coarse flakes was more challenging than that of the fine powder, and, therefore, more HCl was added, reaching a glucosamine protonation level of 82%. The purpose of this preliminary study was to assess whether the chitosan protonation level impacts product performance. Based on the existing literature, we hypothesized that formulations containing higher concentrations of HCl would be more acidic, which would interfere with the solidification of the product, which occurs through clotting of the PRP component, a blood-derived material.

## 2. Materials and Methods

### 2.1. Manufacturing of the Chitosan Freeze-Dried Product

High-molecular-weight chitosans (ChitoClear, Primex, Siglufjordur, Iceland) were subjected to deacetylation with sodium hydroxide and depolymerization with nitrous oxide. Three lower-molecular-weight chitosans were obtained and characterized by nuclear magnetic resonance (NMR) spectroscopy (for degree of deacetylation) [10] and size-exclusion chromatography/multi-angle laser light scattering [11] (for molar mass) (Table 1). Chitosans were solubilized in HCl (Sigma, Oakville, ON, Canada), with HCl concentration adjusted to obtain between 60% and 95% protonation of the chitosan amino groups (Table 1). In addition, the chitosans were first solubilized at 80% protonation, and then NaOH (Sigma, Oakville, ON, Canada) was slowly added to the cold chitosan solution while stirring in order to reach the equivalent of 60% protonation of the chitosan amino groups. Trehalose (Pfanstiehl, Waukegan, IL, USA) (1% *w*/*v* final concentration) and calcium chloride (Spectrum, New Brunswick, NJ, USA) (42 mM final concentration) were added to the chitosan solutions to act as a lyoprotectant and PRP solidification agent, respectively. pH of the chitosan solutions was measured. Solutions were then dispensed in individual vials (3 mL into 10 mL glass vials) for freeze-drying as follows: (1) ramped freezing to −40 °C in 1 h then isothermal 2 h at −40 °C, (2) −40 °C for 48 h at 100 millitorrs and (3) ramped heating to 30 °C in 12 h then isothermal 6 h at 30 °C, at 100 millitorrs.

### 2.2. Isolation of Platelet-Rich Plasma

Citrated sheep blood (Cedarlane, Burlington, ON, Canada) was centrifuged at 800× *g* for 10 min to collect the plasma, buffy coat and a small portion of erythrocytes. These were then centrifuged at 600× *g* for 20 min to separate PRP from the platelet-poor plasma. On average, the PRP contained 4.7 × 10^12^/L erythrocytes (0.6× that of the blood), 21.8 × 10^9^/L leukocytes (4× that of the blood) and 1225 × 10^9^/L platelets (3× that of the blood).

### 2.3. Preparation of Ortho-R (Chitosan–PRP)

A total of 3 mL PRP was pipetted into each chitosan vial and immediately shaken vigorously for 10 s by hand. pH of the chitosan–PRP solutions was measured. Recalcified PRP controls were prepared by recalcifying PRP with calcium chloride at 42 mM final concentration and shaking vigorously for 10 s by hand. Cakes prepared with chitosans 1 and 3 were tested once, and cakes prepared with chitosan 2 were tested twice.

### 2.4. Assessment of Coagulation Kinetics with Thromboelastography

A total of 360 µL of each formulation was loaded immediately after mixing into a thromboelastograph Model 5000 (Haemoscope Corp, Niles, IL, USA). Testing was performed at 37 °C and was allowed to proceed until maximal amplitude was reached. The clot reaction time (R) is the time elapsed between assay initiation until a clot starts to form. On the Model 5000 TEG tracings, this is when the branches have diverged by 2 mm. The maximal amplitude (MA) is the maximal overall width in mm between the 2 diverging branches and corresponds to the maximal attainable clot strength.

### 2.5. Assessment of Clot Homogeneity with Histology

Solubilized formulations were pipetted into glass tubes pre-heated at 37 °C. The clots were fixed in 10% neutral buffered formalin after 1 h. Fixed clots were embedded in paraffin, sectioned at 5 µm thickness and stained with Cibacron Brilliant Red (Glentham Life Sciences, Corsham, UK) and iron hematoxylin (Sigma, Oakville, ON, Canada) [12]. Stained sections were scanned with a Hamamatsu Nanozoomer RS (Hamamatsu City, Japan) using a 40× objective, and images were exported with NDPview software version 2.

### 2.6. Statistical Analysis

All statistical analyses were performed with SAS Enterprise Guide 7.1 and SAS 9.4. Data in the Results section are presented as average ± SD. Data in the figures are presented as median (line); Box: 25th and 75th percentile; Whisker: Box to the most extreme point within 1.5 interquartile range. The One-Way ANOVA task in the SAS Enterprise Guide was used to compare the different groups with post hoc Tukey analysis to look at pairwise differences. Correlations between thromboelastography data and pH were analyzed by calculating the Pearson correlation coefficients (r). *p* values *p* < 0.05 were considered significant.

## 3. Results

### 3.1. Protonation Level of the Chitosan Amino Groups in the Freeze-Dried Product Affects Chitosan–PRP Coagulation Kinetics and Distribution of Chitosan in the Chitosan–PRP Clots

**Figure 1:**
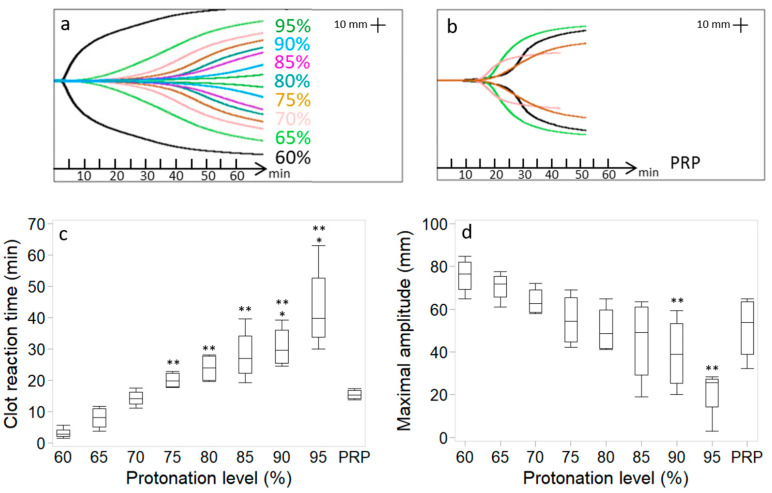
Chitosan–PRP TEG curves of cakes prepared with chitosan 3 (DDA 82.7% *M*_n_ 39 kDa) using different protonation levels (**a**). TEG curves from four different recalcified PRP controls (**b**). Clot reaction time (**c**) and clot maximal amplitude (**d**). Data (n = 4 for each) are presented as median (line); Box: 25th and 75th percentile; Whisker: Box to the most extreme point within 1.5 interquartile range. * *p* < 0.05 versus PRP. ** *p* < 0.05 versus 60% protonated.

Clot reaction time increased with protonation levels (Figure 1), ranging from 3.2 ± 1.7 min at 60% protonation to 43.2 ± 14.1 min at 95% protonation versus 15.4 ± 1.6 min for recalcified PRP. From 75% protonation on, clot reaction time was significantly longer than for 60% protonation (>20.1 ± 2.6 min versus 3.2 ± 1.7 min). From 90% protonation on, clot reaction time was significantly longer than for the PRP control (>30.8 ± 6.7 min versus 15.4 ± 1.6 min). Clot maximal amplitude decreased with protonation levels (Figure 1), ranging from 75.7 ± 8.5 mm at 60% protonation to 20.8 ± 11.9 mm at 95% protonation versus 51.2 ± 15.2 mm for recalcified PRP. Clot maximal amplitude was significantly lower from 90% protonation compared to 60% protonation (<39.4 ± 17.4 mm versus 75.7 ± 8.5 mm).

**Figure 2:**
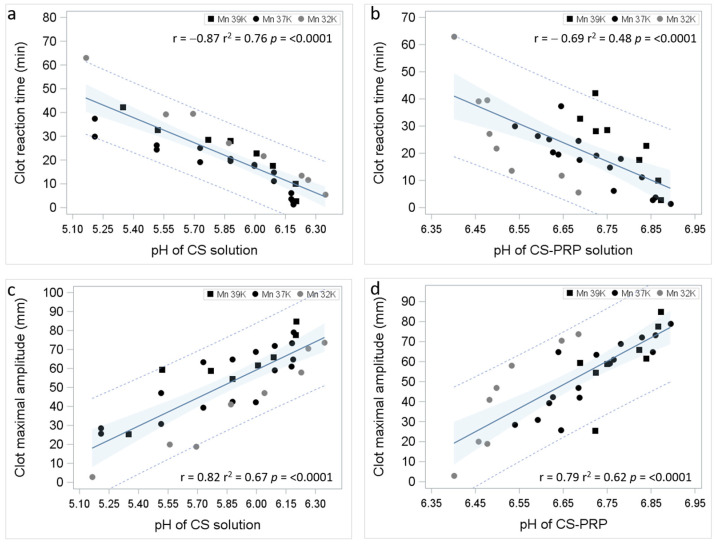
Regression plots showing 95% prediction limits (dashed lines), 95% confidence limits (blue shading), Pearson correlation coefficients r and corresponding *p* values between TEG data and pH of chitosan solution prior to freeze-drying (**a**,**c**) and TEG data and pH of chitosan–PRP mixtures (**b**,**d**).

Strong significant negative correlations were observed between clot reaction time and pH of the chitosan solutions prior to freeze-drying and the pH of the chitosan–PRP mixtures (Figure 2). Strong significant positive correlations were observed between clot maximal amplitude and pH of the chitosan solutions prior to freeze-drying and the pH of the chitosan–PRP mixtures (Figure 2). 

**Figure 3:**
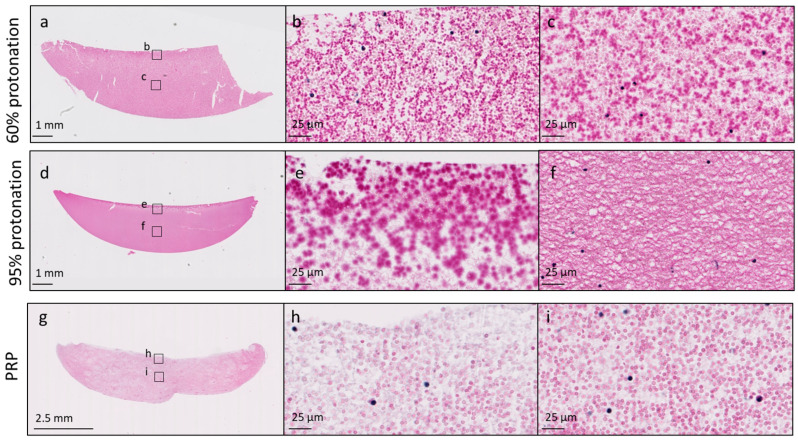
Cibracon Brilliant Red/iron hematoxylin-stained paraffin sections of chitosan–PRP clots prepared with chitosan 3 (DDA 82.7% *M*_n_ 39 kDa) at 60% protonation (panels (**a**–**c**)) and 95% protonation (panels (**d**–**f**)) levels. A recalcified PRP control clot is shown in panels (**g**–**i**). Chitosan is stained dark pink, erythrocytes are stained pale pink and leukocytes are stained black. Slides were scanned with a 40× objective, and high-magnification images were exported at 80× zoom. Panels (**b**,**e**,**h**) were acquired at the surface of the clots, and panels (**c**,**f**,**i**) were acquired in the middle of the clots.

Chitosan distribution was homogeneous in chitosan–PRP clots that were prepared at 60% and 65% protonation levels (Figure 3). As protonation levels increased, chitosan tended to accumulate toward the surface of the chitosan–PRP clots (Figure 3).

### 3.2. Changes in Coagulation Kinetics, Clot Strength and Chitosan Distribution Induced by High Protonation of the Chitosan Amino Groups Are Partially Reversible

**Figure 4:**
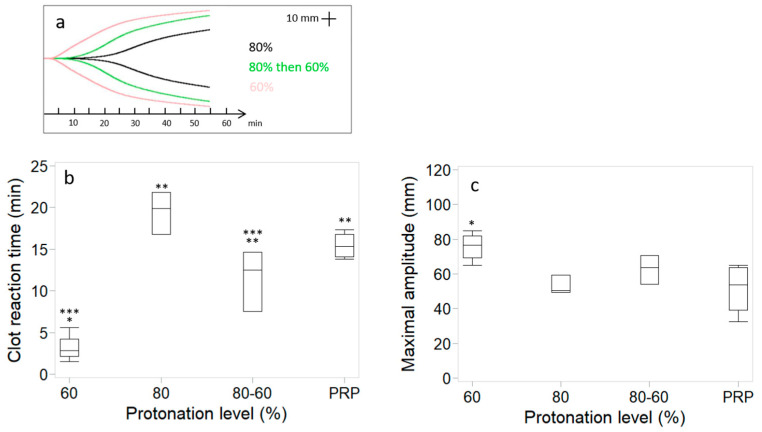
Chitosan–PRP TEG curves of cakes prepared with chitosan 2 (DDA 81.7% *M*_n_ 37 kDa) using 60% and 80% protonation levels and 80% then back to 60% by addition of NaOH (**a**). Clot reaction time (**b**) and clot maximal amplitude (**c**). Data (n = 3 for 80% protonation and 80% back to 60% and n = 4 for 60% protonation and PRP) are presented as median (line); Box: 25th and 75th percentile; Whisker: Box to the most extreme point within 1.5 interquartile range. * *p* < 0.05 versus PRP. ** *p* < 0.05 versus 60% protonated. *** *p* < 0.05 versus 80% protonated.

NaOH was added to chitosan solutions solubilized at 80% protonation level in an attempt to increase pH and improve coagulation kinetics (Figure 4). As expected, the pH was higher for the chitosan solution to which NaOH was added than for the chitosan solution solubilized at 80% protonation level (6.2 ± 0.1 versus 5.8 ± 0.1). Clot reaction time was 19.5 ± 2.5 min for the chitosan solutions solubilized at 80% protonation level and decreased significantly to 11.5 ± 3.6 min when NaOH was added, a value similar to that of recalcified PRP (15.4 ± 1.6 min), but significantly higher than chitosan solutions solubilized at 60% protonation level (3.2 ± 1.7 min). Clot maximal amplitude was 52.9 ± 8.3 mm for the chitosan solutions solubilized at 80% protonation level, lower than for the chitosan solutions solubilized at 60% protonation level (75.7 ± 8.5 mm). The addition of NaOH increased clot maximal amplitude to 62.8 ± 8.3 mm.

**Figure 5:**
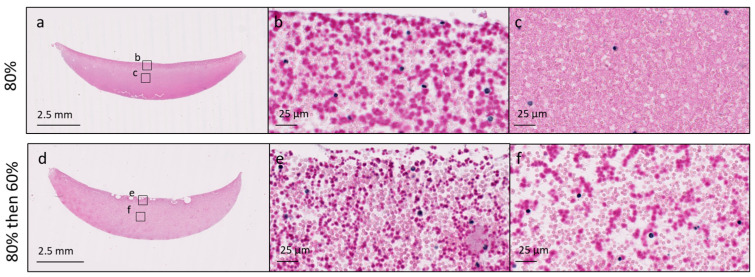
Cibracon Brilliant Red/iron hematoxylin-stained paraffin sections of chitosan–PRP clots prepared with chitosan 2 (DDA 81.7% *M*_n_ 37 kDa) at 80% protonation (panels (**a**–**c**)) and 80% then back to 60% by addition of NaOH (panels (**d**–**f**)). Chitosan is stained dark pink, erythrocytes are stained pale pink and leukocytes are stained black. Slides were scanned with a 40× objective, and high-magnification images were exported at 80× zoom. Panels (**b**,**e**) were acquired at the surface of the clots, and panels (**c**,**f**) were acquired in the middle of the clots.

Chitosan was distributed at the surface of the chitosan–PRP clots solubilized at 80% protonation level (Figure 5). The addition of NaOH ensured that chitosan was distributed homogeneously throughout the chitosan–PRP clots (Figure 5).

## 4. Discussion

Our starting hypothesis that protonation level would impact the solidification of the Ortho-R product was confirmed. For use in the context of surgical repair, we believe that it would be desirable to have a product with lower protonation levels. At 60–65% protonation, the formulation solidifies faster than recalcified PRP by itself, and the resulting CS-PRP hybrid clots are much stiffer than PRP clots. Faster solidification post-application would decrease operating room time and yield implants that are more resistant to mechanical forces. In addition, the homogeneous distribution of the chitosan component within the CS-PRP hybrid clots may be important for optimal biodegradability. Nevertheless, the Ortho-R product produced with 82% protonation was used successfully in the rotator cuff sheep pivotal study [9], so the protonation level may not have that much of an impact on repair responses once implanted in vivo.

Hemostasis and coagulation involve several overlapping and interlinked steps [13]: (1) activation of platelets, aggregation and formation of a platelet plug, (2) enzyme-catalyzed generation of thrombin and (3) conversion of fibrinogen into fibrin, cross-linking and formation of a fibrin/platelet clot. The generation of thrombin occurs in stages: (1) small amounts of thrombin are produced in tissue factor (TF)-bearing cells during the initiation phase through the activation of the Factor VIIa/TF complex and Factor Xa; (2) this thrombin then activates Factors Va, VIIIa and XIa on the surface of activated platelets during the amplification stage; (3) finally, large amounts of thrombin are created during the propagation phase, through the assembly of the tenase (Factor IXa/Factor VIIIa) and prothrombinase (Factor Xa/Factor Va) complexes on the surface of activated platelets. Inhibition or disruption of one or several of these steps would prevent hemostasis and coagulation.

Acute traumatic coagulopathy (ACT) is characterized by systemic anti-coagulation and hyperfibrinolysis [14,15,16,17,18], and hypothermia and metabolic acidosis have been shown to aggravate this hypocoagulable state. In vivo swine models have been developed to better understand the effect of acidosis and hypothermia on coagulopathies, as trauma patients often present with this “lethal triad”. Darlington and colleagues induced acidosis in swine through HCl infusion or hemorrhage/hypoventilation [19,20]. Fibrinogen concentration and platelet counts in blood decreased. Standard coagulation tests and thromboelastography showed that acidosis compromised coagulation function. In a series of studies by Martini and colleagues [21,22,23,24], swine were subjected to acidosis with HCl infusion, hypothermia or both. Platelet counts in blood were decreased, and spleen bleeding time was increased by acidosis, hypothermia and the combination of both treatments. Acidosis and hypothermia inhibited coagulation through different mechanisms. Acidosis was found to inhibit the propagation phase of thrombin generation, while hypothermia inhibited the initiation phase of thrombin generation. Fibrinogen metabolism was also affected differently by acidosis and hypothermia. Acidosis accelerated fibrinogen degradation, while hypothermia diminished fibrinogen synthesis, both of which led to a decrease in fibrinogen availability. In summary, it has been well established that blood coagulation is impaired by acidosis through different mechanisms in vivo.

Laboratory studies dating back decades showed that blood thrombin time, prothrombin time, partial thromboplastin time and plasma recalcification time all increase in a progressive manner with acidosis [25,26]. In the current study, we found that clot reaction time (R) was significantly increased by acidosis. This is in agreement with previous studies investigating the effect of pH on blood and PRP clotting in vitro [27,28,29]. Since R corresponds to the initiation phase of clotting that is driven by plasma coagulation enzymes, this suggests that the activity of the coagulation factors was compromised by acidification in our study. Clot formation time (k) increased and alpha angle (α) decreased with acidosis in the current study, but, in contrast to previous in vitro studies [27,28,29,30], our results did not reach statistical significance due to high variability. Clot formation time (k) and alpha angle (α) are reflective of the amplification and propagation phases of thrombin generation and measure the speed at which fibrin builds up and polymerizes. They are thus strongly dependent on the activity of coagulation enzymes and the availability of fibrinogen and activated platelet surfaces. Contrary to what was previously described in swine models [19,20,21,22,23,24], we feel that it is unlikely that fibrinogen levels and platelet counts were decreased by acidosis in our in vitro system, and so impairment of coagulation enzyme activity probably caused these changes in coagulation kinetics. Other authors have shown that a small decrease in pH can reduce the activity of several proteases comprising the coagulation cascade: the activities of Factor VIIa (FVIIa), of the FVIIa/Tissue Factor (FVIIa/TF) complex and of the prothrombinase (Factor Xa/Factor Va) complex decreased significantly by 90%, 55% and 70%, respectively, when pH is reduced from 7.4 to 7.0 [31]. It is not unreasonable to hypothesize that other coagulation enzymes, such as the cross-linker Factor XIII, would also be less active in acidic conditions. Since CS–blood mixtures solidify through coagulation mechanisms that involve thrombin generation, platelet activation and fibrin polymerization [32], it is not surprising that the coagulation of CS-PRP mixtures would be impaired by decreases in pH.

We also found that acidosis caused a significant decrease in clot maximal amplitude (MA) in the current study. This could be partly explained by the alteration of chitosan distribution within the clots with acidosis. As pH decreased, chitosan accumulated toward the surface of the clots instead of being distributed throughout. This could have increased vacuolization within the body of the clot, which would result in poor attachment between the forming clot and cup and pin, and once attached, the tensile strength would be decreased within the body of the clot. However, several studies have reported decreased clot strength when blood or PRP were acidified [27,28,29], suggesting that acidosis can affect the overall stability of the clot regardless of whether biomaterials are present or not within the clot. Clot maximal amplitude is mainly dependent on the interactions of platelets and fibrin through GPIIb-GPIIIa receptors. Platelet activation, conversion of fibrinogen into fibrin and cross-linking through enzymatic activity are therefore critical steps in attaining a high clot maximal amplitude (MA). As stated above, we believe that platelet counts and fibrinogen concentration were most likely not decreased by acidosis in this in vitro model. However, acidosis has been shown to decrease platelet activation and aggregation in a process that is thought to involve pH effects on calcium influx into the platelets and inhibition of αIIbβ3 integrin conformational changes [25,26,30,33,34]. A decrease in platelet activation and binding to fibrin could have happened here in the most acidic conditions. 

The main limitation of our study is that we did not investigate the mechanisms by which coagulation of CS-PRP was impaired by acidosis. In future studies, the kinetics of thrombin generation, platelet activation and Factor XIII activation could be assessed as previously described for CS–blood mixtures [32]. Increasing the number of repetitions for each condition would also be recommended due to the inherent variability of biological samples.

We found that adding NaOH to the chitosan mixtures post-solubilization but pre-freeze-drying to increase pH can partially restore the coagulation kinetics of the product. Interestingly, although impaired platelet aggregation induced by acidosis was found to be reversible by the addition of NaOH [26], Darlington, Martini and colleagues showed that pH correction alone is not sufficient to correct acidosis-induced coagulation impairment [19,20,21,22,23,24]. Unfortunately, this manufacturing step would be difficult to implement during high-scale production since the chitosan solution needs to be constantly cooled while stirring and the NaOH added drop by drop in order to prevent local precipitation of chitosan. We therefore modified the manufacturing process to incorporate a 48 h chitosan hydration step in water prior to adding the HCl. The CMO produced a second lot of Ortho-R product using that process and was able to solubilize the coarse flakes of chitosan using a 60% protonation level. This lot is currently being used in a rotator cuff repair randomized controlled trial comparing Ortho-R-augmented surgical repair to the standard of care. In the future, we will consider implementing a cryogenic grinding step in the chitosan manufacturing process in order to reduce the coarse flakes into a fine powder that can be easier to solubilize. Conventional grinding, which produces heat, may not be appropriate for heat-sensitive chitosan.

In conclusion, careful control of manufacturing processes is critical, and it is important to consider the impact of each manufacturing step on product performance. In the case of the Ortho-R (ChitogenX Inc., Kirkland, QC, Canada) combination drug–biologic product, chitosan solubilization at 60–65% protonation of the glucosamine units on the chitosan chains appears to be optimal.

## Figures and Tables

**Table 1:** HCl concentrations used to solubilize the different chitosans.

Chitosan Properties	Chitosan 1 DDA 84.8% *M*_n_ 32 kDa	Chitosan 2 DDA 81.7% *M*_n_ 37 kDa	Chitosan 3 DDA 82.7% *M*_n_ 39 kDa
60% protonation	30 mM HCl	29 mM HCl	29 mM HCl
65% protonation	33 mM HCl	31 mM HCl	32 mM HCl
70% protonation	35 mM HCl	34 mM HCl	34 mM HCl
75% protonation	38 mM HCl	36 mM HCl	37 mM HCl
80% protonation	41 mM HCl	39 mM HCl	39 mM HCl
85% protonation	43 mM HCl	41 mM HCl	42 mM HCl
90% protonation	46 mM HCl	44 mM HCl	44 mM HCl
95% protonation	48 mM HCl	46 mM HCl	47 mM HCl
80% protonation then 60%	41 mM HCl then 11 mM NaOH	39 mM HCl then 10 mM NaOH	39 mM HCl then 10 mM NaOH

DDA: degree of deacetylation; *M*_n_: number average molar mass.

## Data Availability

Dataset available upon request from the authors.
